# The psychological outcomes of COVID-19 affected the pandemic-after risk perceptions of nurse clinicians: a latent profile analysis

**DOI:** 10.1017/gmh.2022.13

**Published:** 2022-03-08

**Authors:** Yin Qianlan, Hou Tianya, Li Wei, Gao Jia, Ni Chunyan, Zhao Wei, Lian Bin, Li Huifen, Dong Wei, Deng Guanghui, Jia Yan

**Affiliations:** 1 Department of Naval Aviation & Operational Psychology, Navy Medical University, Shanghai, China; 2 Suzhou Science & Technology Town Hospital, Gusu School, Nanjing Medical University, Suzhou, China; 3 Changshu Hospital Affiliated to Nanjing University of Traditional Chinese Medicine, Changshu, China

**Keywords:** COVID-19 pandemic, latent profile analysis, nurse clinicians, psychological outcomes, risk perceptions

## Abstract

**Background:**

Risk perception among nurses after the COVID-19 pandemic is a crucial factor affecting their attitudes and willingness to work in clinics. Those with poor psychological status could perceive risks sensitively as fears or threats that are discouraging. This article aimed to determine whether psychological outcomes, including post-traumatic stress disorder (PTSD), depression, anxiety, and insomnia, following the COVID-19 pandemic were differentially related to the risk perceptions of nurses working in clinics and increased perceived risk.

**Method:**

The participants were 668 nurse clinicians from five local hospitals. Risk perceptions and psychological outcomes were measured by adapted questionnaires via the Internet. Latent profile analysis (LPA) identified subgroups of individuals who showed similar profiles regarding the perceived risks in nursing. Multinomial regression and probit regression were used to examine the extent to which sociodemographic and psychological outcomes predicted class membership.

**Results:**

LPA revealed four classes: groups with low-, mild-, moderate-, and high-level risk perceptions. Membership of the high-level risk perception class was predicted by the severity of psychological outcomes. Anxiety significantly accounted for a moderate increase in risk perceptions, while the symptoms of insomnia, depression, and PTSD accelerated the increase to the high level of risk perception class.

**Conclusions:**

By classifying groups of nurse clinicians sharing similar profiles regarding risk perceptions and then exploring associated predictors, this study shows the psychological outcomes after COVID-19 significantly impacted pandemic-associated risk perceptions and suggests intervening in nurses' psychological outcomes while simultaneously focusing on work-related worries is important following the outbreak of COVID-19.

## Introduction

Pandemic restrictions have imposed both hardships and inconveniences in everyday life. People have entered a new ‘normal’ life with masks and social distances, hoping for the arrival of a vaccine. However, potential risks are everywhere and cannot be ignored. As the largest group of health care workers, nurses are on the frontline of the health care system's response to COVID-19 and experience several potential risks during nursing operations (WHO, 2020*a*). Due to nurses providing care in close physical proximity, they experience an increased risk of developing the disease because they are often directly exposed to viruses. Unfortunately, they have no choice but to confront inherent threats; otherwise, an unescapable tragedy would happen.

### Risk perceptions of nurse clinicians

Nurses' risk perceptions are a crucial factor affecting their work attitudes, willingness, quality of nursing care, job satisfaction, and prosocial behaviors. The notion of perceived risk to health professionals during pandemics has been explored in the literature (Koh *et al*., 2011), yet fewer studies report data about nurse clinicians, as distinct from other health professionals. Nurses' risk perceptions refer to their knowledge, feelings, and understanding of risk factors or risk characteristics in the profession. Generally, it is a suitable mental and behavioral reaction for nurses. To some extent, individuals must be aware of the disease, acknowledge their vulnerability to it, and safeguard themselves with prevention methods or treatment (Homko *et al*., 2008). Referring to the health belief model (Rosenstock, 1974; Janz and Becker, 1984; Champion and Skinner, 2008), risk perception is deemed personal beliefs about the likelihood of suffering a disease. Individuals who perceive a high level of susceptibility to a particular disease will adopt necessary measures to reduce the risk of developing it; individuals with low perceived susceptibility may deny that they are at risk for contracting a particular illness (Janz and Becker, 1984). In other words, risk perceptions could prevent people from engaging in risky behaviors. For example, people show various levels of risk perceptions that could predict their preventive behaviors, and with risk exaggerators describe high preventive behaviors (Wang *et al*., 2021). However, for those occupied in high-risk work environments such as nurse clinicians, the risk perceptions of health problems or other potential dangers would, either subconsciously or consciously, disrupt their work or discourage them. Therefore, risk perceptions about the pandemic can play a critical role in medical practices.

### The risk perceptions of nurse clinicians for COVID-19

During the pandemic, nurses' knowledge of the disease was messy, and their attitudes and practices were disrupted; therefore, their risk perceptions were affected. Being infected, transmitting the infection to family members, acknowledging the stigma about the vulnerabilities of their job, and restrictions on personal freedom have been reported as key concerns in previous studies (Chiang *et al*., 2007; Seale *et al*., 2009; Hope *et al*., 2011; Koh *et al*., 2012). Complicating the situation for nurses working in clinics during COVID-19 are the logistical issues related to the supply of personal protective equipment and shortages of other necessary resources to support service delivery (Xie *et al*., 2020). For nurses who remain in clinical practice, an obvious impact relates to psychosocial ramifications. As a repeated cross-sectional study on frontline workers in a COVID-19 hub-hospital reported, during the first wave, workload and compassion fatigue increased (Magnavita *et al*., 2020); with the development of the crisis, the heavy workload, isolation at work, uncertainty about safety procedures, and the abrupt decline in the time devoted to meditation and relaxation evolved to become prevalent causes of occupational stress (Magnavita *et al*., 2021*a*); one year after the baseline, an increased workload, isolation at work and in social life, lack of time for physical activity and meditation, and compassion fatigue are still reported in health workers and coexist with new worries for justice in safety procedures (Magnavita *et al*., 2021*b*). However, there is a significant variation in the extent to which nurses perceive risk in the clinical environment or events. Surveys have reported that fewer people have a high perception of risk related to the virus itself, while more people indicate stress caused by concerns related to pandemic-associated responses (Cornelia *et al*., 2020). Moreover, some nurses on the frontline may show fewer worries and fears for their safety since they believe in the effectiveness of their protective equipment, while many nurse clinicians, not at the frontline, that encounter issues with ineffective and insufficient medical protection, fidget and become discomposed (Risti *et al*., 2020). Risk perception is a mental psychological construct that is subject to cognitional, emotional, sociocultural, political, and personal variabilities (Linden, 2014, 2015; Samadipour *et al*., 2020). Moreover, potential risks are not all concurrent. This has led to an increased interest in understanding how nurse clinicians perceived their risks for COVID-19. Are there subgroups of nurse clinicians who present with similar patterns of perception co-occurrence? If so, do these subgroups share characteristics that can be identified as indicators for their risk perceptions patterns and extent? Notably, the extent of risk perceptions closely related to the nurses' motivation for their clinic work and practice could have important implications for health care defense. Hence, assessing the risk perceptions of nurse clinicians for COVID-19 and its factors is indispensable.

### Psychological impacts of COVID-19 on nurses

In addition, the pandemic has affected nurses' risk perceptions through their mental and psychological constructions. Individuals' emotions and instincts affect their perceptions, and they acquire these perceptions through observations, analysis of circumstances, and scientific consultations (Aakko, 2004). It is well-established that the pandemic and its restrictions have led to psychosis and have harmed the well-being and mental health of many nurses without exception. Worse still, people already experiencing distress are affected even more negatively (Osofsky *et al*., 2020; Yancy, 2020). Risk perception has its roots in psychological constructures and lies in continuity between no risk and high risk, positively and negatively affecting the decision-making (Chaiklin, 1987; Aakko, 2004; Kendra and George, 2010). Furthermore, the cognitive and decision-making abilities of nurses are influenced by their own emotions (Shirey, 2010; Standing, 2010; Radtke *et al*., 2012; Magnavita *et al*., 2020), which informs us of the potential relationship between nurses' psychological status and their risk perceptions. Initial psychological studies of COVID-19 showed that symptoms of depression and anxiety were present in most health care workers (Magnavita *et al*., 2020; Que *et al*., 2020; Santamaria *et al*., 2020). A systematic review and meta-analysis of articles related to COVID-19 identified poor sleep quality, stress, psychological distress, insomnia, post-traumatic stress disorder (PTSD), anxiety, and depression (Kontoangelos *et al*., 2020). Moreover, a one-year prospective study also found that 73% of investigated doctors reported high levels of distress, 64% reported depression, 28% reported sleep problems, and 25% reported anxiety (Magnavita *et al.*, 2021*b*). Notably, stress and anxiety can evolve into acute stress disorder and precede chronic PTSD. Depressive symptoms are likely to follow chronic unresolved anxiety. Anxiety and depressive symptoms may include constant fear, excessive worrying, poor concentration, disturbances in sleep or appetite, low energy or fatigue, and decreased motivation (Skarl, 2015). To our knowledge, worsened psychological states could impair cognitive processes and individual characteristics, which are elements of risk perceptions. However, the psychological impacts of COVID-19 on nurse clinicians and their association with nurses' risk perceptions are scarcely reported. Therefore, advancing our understanding of the relationship between the prevalent psychological outcomes (including PTSD, depression, anxiety, and insomnia) and characteristics of risk perceptions has significant clinical references.

### The present study

Understanding the factors that impact the risk perceptions of nurse clinicians is essential to inform future workforce policy and institutional responses. Hence, this study sought to extend on previous work to examine the relationship between the psychological impact of COVID-19 and the pandemic-associated risk perceptions of nurse clinicians. We proposed our hypotheses that poor psychological responses to COVID-19 in nurses could relate to high-risk perceptions and that people with a similar level of risk perceptions may complain of psychological problems to varying degrees. Additionally, risk perception is one of the vital factors related to nurses' willingness to perform clinical care (Chaffee, 2009; Khalid *et al*., 2021). Therefore, we speculated that poor psychological responses and high-risk perceptions indicated a high likelihood of being discouraged to perform clinical care duties and that immediate intervention should be considered. Meanwhile, previous studies have identified individual characteristics, and sociodemographic and exposure-related variables that differentially predict risk perceptions (Rosenstock, 1974; Champion and Skinner, 2008), and our analyses also considered these factors.

## Methods

### Participants and procedures

This cross-sectional study was conducted in Jiangsu Province, China. Data were collected in early January 2021, one year after the outbreak of the COVID-19 infection. Nurse clinicians from five local hospitals in Jiangsu Province were recruited with the help of nursing clinical directors, who distributed the survey website in their working groups. Therefore, a random cluster sampling was used in the study. All the hospitals had admitted COVID-19 confirmed or suspected patients during the peak of the epidemic and no COVID-19 patients at the time of the survey. Finally, a total of 707 nurses voluntarily participated in the study and finished the survey. The inclusion criteria for valid questionnaires were as follows: the nurse clinician should (a) be a nurse in the hospital in the COVID-19 pandemic year (2020–2021); (b) have no history of mental disorder including depression or anxiety disorders diagnosed before the COVID-19; and (c) be at least 18 years old. These three questions were presented on the first page of our online survey, and only those who answered ‘yes’ to these questions could continue to the next page. The exclusion criterion included those with missing values in any studied variables. Finally, 668 participants were included in the analysis, and their working experiences were also collected. Ethical approval was obtained from the Second Military Medical University before the initiation of the research project. Before the online survey, all participants received informed written consent and were told that they could withdraw at any time. Additionally, anonymity and confidentiality were assured.

### Measures

#### Demographic questionnaire

This questionnaire included question information regarding gender, age, education level, marriage, departments. Additionally, considering COVID-19 exposure, we also asked whether they contacted the confirmed patients.

#### Nurses' risk perceptions questionnaire

The questionnaire for risk perceptions was referred to the questionnaire revised by the Chinese researchers (Zhang *et al*., 2016), including 26 questions pertaining to six domains of risk: personal safety risk (R1–R5; e.g. *worries about fights*, *unsafe night commute*, *disgruntle or manic patients*); physical function risk (R6–R9; for instance, *occupational diseases*, *gastritis*, *or varicose veins*), occupational exposure risk (R10–R13; e.g. *infection or work environment*), psychosocial evaluation risk (R14–R18; *patients' complains*, *colleagues' judgment or superiority of doctors*), organizational risk (R19–R22; e.g. *security hole*, *shortage of staff or facilities*), and time pressure risk (R23–R26; e.g. *less time for family union or less leisure time*). The frequency of the worries, equal to the potentiality of perceived risk, is divided into five grades from ‘never’ to ‘almost always’ and then assigned 1–5 points in turn. The higher the score for each item indicated the higher awareness of the risk. The *α* coefficients for the subscales ranged from 0.908 to 0.970.

#### Questionnaires for psychological outcomes

Psychiatric questionnaires were conducted to measure the psychological outcomes. The impact of Event Scale-Revised (IES-R) was adopted as it is not diagnostic for PTSD but an appropriate instrument to measure the subjective response to a specific traumatic event in the senior population, as well as a total subjective stress IES-R score recognized as the proper self-report tools to assess post-traumatic stress after COVID-19 (Christianson and Marren, 2012; Chew *et al*., 2020). IES-R has three subscales (Intrusion, Avoidance, and Hyperarousal), which are closely affiliated with PTSD symptoms. Items have all turned the focus on the event of the COVID-19 pandemic outbreak. Participants were required to rate their distress brought by COVID-19. Simultaneously, their current sleep quality, anxiety, and depression were also measured respectively by Insomnia Severity Index (ISI) (Bastien *et al*., 2001), The Generalized Anxiety Disorder Scale (GAD-7) (He *et al*., 2010), and Patient Health Questionnaire (PHQ-9) (Kroenke *et al*., 2001). Likewise, for our purpose of evaluating the psychological fallout of the COVID-19 pandemic, we added the sentence – ‘Comparing to before, this pandemic year makes you feel…’ – to the original symptom items. The total score of each questionnaire evaluated the psychological state during the recent year.

### Statistical analysis

Latent profile analysis (LPA) was conducted in Mplus version 8.3 (Muthén and Muthén, 1998–2017) to identify and describe unobserved classes of risk perceptions among HCWs (Hagenaars and Mccutcheon, 2002). LPA is a person-centered methodological approach that helps to elucidate population heterogeneity within observed data through the identification of underlying subgroups of individuals, thus allowing the empirical examination of a distinct complex construct. The modified approach to modeling was applied as ratings of HCW's risk perceptions were scored as continuous variables, which could be appropriately tested by the method (Vermunt, 2010). Since the exact number of latent classes representing risk perceptions was unknown, an exploratory approach was used firstly, which started with the most one-class model and fitted successive models with increasing numbers of classes. Each latent class solution was replicated 20 times beginning at random starting values. This method included a close examination of item loadings and model fit indices for estimated latent classes (Vermunt, 2010; Asparouhov and Muthén, 2014). The final number of classes was determined based on the conceptual meaning, smallest estimated class proportions, entropy, and statistical model fit indices such as the Akaike Information Criterion (AIC), Bayesian Information Criterion (BIC), and adjusted BIC (Nylund *et al*., 2007; Asparouhov and Muthén, 2014). Latent classes with <5% of the total sample were not considered due to the possibility of class over-extraction in the presence of non-normal data and poor generalizability (Bauer and Curran, 2003; Grimm, 2017). Maximum likelihood estimation with robust standard errors incorporating all available data was used to deal with missing data and to estimate parameters. Mplus accounted for the complex survey design by correcting the standard errors and *χ*^2^ tests of model fit (Hagenaars and Mccutcheon, 2002).

Descriptive statistics for the demographic characteristics and psychological reactions to COVID-19 of the study sample and comparisons across groups were analyzed using SPSS version 26.0. Meanwhile, the distribution of identified classes and their associations with psychological reaction and demographic characteristics were examined by the one-way analysis of variance (ANOVA) and *χ*^2^ analysis. Further, the Multinomial Logit Model was built with the Wald tests for the effects of covariates on class membership. Regression was performed using the psychological reactions to predict the contingent probability of the identified classes of HCW's risk perceptions. Statistical significance was taken as a two-sided *p* < 0.05.

## Results

### LPA for risk perceptions

**Fig. 1.**
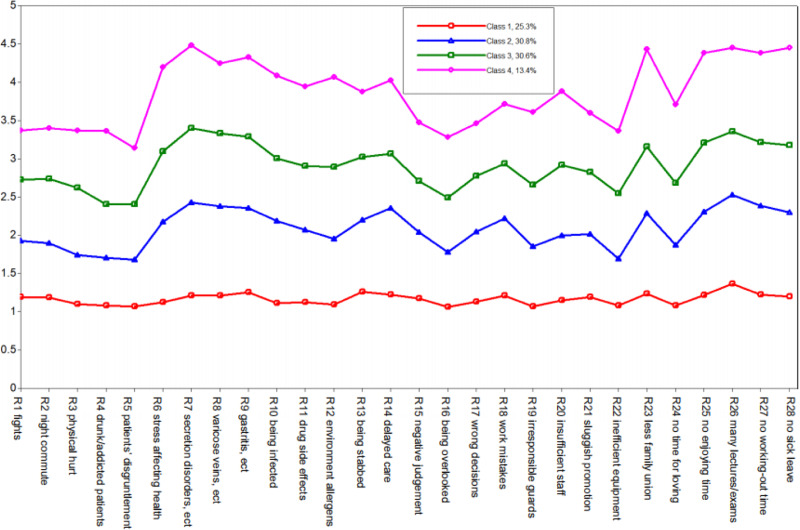
Estimated means of risk perceptions and percentiles for the four-class solution.

Table 1 presents the fit indices for the LPA. Based on fit indices and interpretability of class solutions, a four-class solution was the optimal solution. The five-class solution yielded a slightly lower adjusted BIC and AIC with the bigger value of entropy than the three-class model; however, the value of entropy was not improved and the non-significant Lo–Mendell–Rubin (LMR) test indicated that this model failed to fit the data significantly better than a four-class model. Therefore, a four-class solution was judged as the optimal solution. This solution comprised four classes of nurses with different levels of risk perceptions which could be classified as high (class 4), moderate (class 3), mild (class 2), and low (class 1). Conditional means of each risk for the four-class solution are displayed in Fig. 1. Values >3.5 were considered to represent a high level of perceived risk in the class (i.e. the weighted average rating of this group was estimated to be 3.5 points on the item), values between 3.5 and 2.5 presented a moderate level, values between 2.5 and 1.5 a mild level, and below 1.5 were low-risk level. As can be seen in Fig. 1, all classes presented the same patent for risk perceptions. Class 4 evidenced a high probability of rating high scores for physical function, organizational risks, and time pressure risks, only slightly low in threats from patients to their safety; class 3 and class 2 also showed similar sensitivity to the different risk domains; however, class 1 tended to be insensitive to all risks.

**Table 1.** Goodness-of-fit statistics for 1–6 class solutions

Model	*K*	AIC	BIC	eBIC	Distribution	Probability	Entropy	LMR
1	112	52 921.123	53 425.604	53 069.996				
2	225	42 563.657	43 577.122	42 862.732	236/432	0.353/0.647	0.984	0
3	338	37 760.289	39 282.739	38 209.567	257/155/256	0.385/0.232/0.383	0.979	0
4	451	35 493.758	37 525.192	36 093.238	178/226/111/153	0.266/0.338/0.166/0.229	0.985	0.7379
5	564	34 441.774	36 982.182	35 191.456	97/179/111/142/139	0.145/0.268/0.166/0.213/0.208	0.985	0.7604
6	677	33 753.186	36 802.59	34 653.071	85/51/143/151/140/98	0.127/0.076/0.214/0.226/0.209/0.147	0.984	0.7624

*K*, free parameter; AIC, Akaike information criterion; BIC, Bayesian information criterion; e-BIC, sample size-adjusted Bayesian information criterion; LMR, Lo–Mendell–Rubin.

### Differences between LPA classes

Participant characteristics, COVID-19 related experiences, and mean psychological outcome scores for each of the LPA classes were also presented in Table 2. The *χ*^2^ analysis demonstrated no significant differences between classes in terms of marriages, department, positions, contact history, age, years of being working. However, gender was significantly disparate between classes as women tended to be more worried than men. Notably, a series of one-way ANOVAs indicated that the psychological outcomes were related to the risk perceptions. Post hoc tests showed between-class differences were all significant (*p* < 0.001). In class 4, the average score of IES, ISI, PHQ, and GAD were the highest, while in class 1, they were the lowest, thus indicating the high class scored significantly higher than the lower on each sub-scale.

**Table 2.** Participants characteristics and psychological outcomes one year after COVID-19

	Class 1	Class 2	Class 3	Class 4	Total	*χ*^2^/*F*	*p* value	
Gender						7.996	0.046	
Male	11.20%	4.90%	7.40%	3.30%	7.00%			
Female	88.80%	95.10%	92.60%	96.70%	93.00%			
Marriage						6.246	0.715	
Single	31.40%	26.70%	28.60%	25.60%	28.30%			
Married	64.50%	69.90%	69.50%	68.90%	68.30%			
Divorced	4.20%	3.40%	2.00%	5.50%	3.40%			
Department						32.096	0.057	
Internal	20.10%	28.60%	35.00%	34.40%	29.20%			
Surgical	16.60%	17.50%	22.20%	20.00%	19.00%			
Emergency	8.90%	7.30%	7.90%	7.80%	7.90%			
ICU	4.10%	5.80%	3.90%	5.60%	4.80%			
Obstetrics and gynecology	18.90%	16.50%	12.80%	21.10%	16.60%			
Pediatric	4.70%	2.90%	4.40%	1.10%	3.60%			
Operating	11.20%	6.30%	5.40%	4.40%	7.00%			
Other	15.40%	15.00%	8.40%	5.60%	11.80%			
Position						13.835	0.311	
Nurses	21.30%	15.50%	13.80%	8.90%	15.60%			
Senior nurses	52.70%	55.30%	50.20%	54.40%	53.00%			
Supervisor nurse	4.10%	4.40%	5.40%	4.40%	4.60%			
Position						3.994	0.262	
Yes	3.60%	2.90%	4.90%	7.80%	4.30%			
No	96.40%	97.10%	95.10%	92.20%	95.70%			
Age	25.49 ± 5.94	25.88 ± 5.70	26.73 ± 6.03	26.86 ± 5.95	26.17 ± 5.91	1.938	0.122	
Years of being work	8.07 ± 6.95	8.17 ± 6.05	9.01 ± 6.61	9.33 ± 6.56	8.56 ± 6.53	1.318		0.267
IES	2.11 ± 4.038	8.66 ± 8.91	18.75 ± 14.43	22.22 ± 14.80	11.90 ± 13.30	102.959	0.000	
ISI	2.60 ± 3.43	4.93 ± 4.49	8.59 ± 5.38	11.88 ± 5.95	6.39 ± 5.70	96.046	0.000	
PHQ	2.19 ± 2.66	5.16 ± 3.58	8.03 ± 4.84	12.34 ± 6.12	6.25 ± 5.32	130.767	0.000	
GAD	0.60 ± 1.34	2.79 ± 3.05	4.90 ± 4.16	7.72 ± 5.32	3.54 ± 4.20	94.696	0.000	

IES, The impact of Event Scale-revised; ISI, Insomnia Severity Index; PHQ, Patient Health Questionnaire items; GAD, The Generalized Anxiety Disorder Questionnaire.

### The predictive effects of psychological variables

To examine the long-term effect of COVID-19 on nurses' risk perceptions, a multinominal logistic regression and a probability regression were respectively conducted by using the total score of IES, ISI, GAD, or PHQ as predictors. Due to the near-significant result for gender between classes, we also included this variable as a co-variable in the multinominal logistic regression model with class 1 as the reference class. As Table 3 showed, gender differences were only slightly significant in the comparison between class 4 and class 1; however, psychological outcomes strongly predicted the class of risk perceptions with between-level disparities.

**Table 3.** Multinomial logistic regression predicting class membership using class 1 as reference

Predictor		*B*	s.e.	Exp(B)	95% CI	*p* value
Class 2 compared to class1	Gender (male = 0)	−0.889	0.48	0.411	(0.16–1.05)	0.064
	IES	0.113	0.026	1.119	(1.06–1.18)	0.000
	ISI	0.039	0.037	1.04	(0.97–1.12)	0.292
	PHQ	0.119	0.055	1.126	(1.01–1.26)	0.032
	GAD	0.239	0.087	1.27	(1.07–1.51)	0.006
Class 3 compared to class 1	Gender (male = 0)	−0.78	0.56	0.458	(0.15–1.37)	0.163
	IES	0.175	0.026	1.191	(1.13–1.25)	0.000
	ISI	0.136	0.039	1.145	(1.06–1.24)	0.001
	PHQ	0.19	0.061	1.209	(1.07–1.36)	0.002
	GAD	0.191	0.092	1.21	(1.01–1.45)	0.038
Class 4 compared to class 1	Gender (male = 0)	−1.646	0.797	0.193	(0.04–0.92)	0.039
	IES	0.158	0.028	1.171	(1.11–1.24)	0.000
	ISI	0.188	0.044	1.206	(1.11–1.31)	0.000
	PHQ	0.338	0.068	1.402	(1.23–1.6)	0.000
	GAD	0.177	0.098	1.194	(0.99–1.45)	0.070

IES, The impact of Event Scale-revised; ISI, Insomnia Severity Index; PHQ, Patient Health Questionnaire items; GAD, The Generalized Anxiety Disorder Questionnaire; s.e., standard error; CI, confidence interval.

For further analysis of the predicted value, a regression on the post probability of categorized risk perceptions with psychological outcomes was conducted, results from which were presented in Table 4. This prediction analysis revealed that the score of IES-R significantly increased the possibility of being classified from class 1 to class 2 by 12.3%, from class 1 to class 3 by 19.8%, and from class 1 to class 4 by 6.6%. Similarly, increased scores in ISI and depression significantly elevated the possibility of being classified into the higher risk perceptions. Especially, in comparison, GAD scaling for anxiety only significantly accounted for the increase from low-risk perception to mild (*p* *=* 0.012), but no significance for further elevation. Referred to class 3, increases in PHQ scaling for depression added the possibility of being classified to class 4 by 16.5%. Anxiety existed in all classes and significantly augmented nurses' risk perception into a mild class instead of a moderate one; however, the PTSD after COVID-19, insomnia, and depression would render moderate and high-risk perception in nurses. As a whole, in our samples, we attained that psychological status affected risk perceptions.

**Table 4.** Alternative parameterizations for the categorical latent regression using class 1 as reference

Parameterization	Est	s.e.	Est./s.e.	Possibility increased (%)	*p* value
Class 2 referred to class 1					
IES	0.116	0.029	3.998	12.3	0.000
ISI	0.038	0.04	0.963	3.9	0.335
PHQ	0.124	0.061	2.036	13.2	0.042
GAD	0.247	0.098	2.507	28.0	0.012
Class 3 referred to class 1					
IES	0.181	0.03	6.09	19.8	0.000
ISI	0.139	0.043	3.217	14.9	0.001
PHQ	0.195	0.069	2.846	21.5	0.004
GAD	0.197	0.103	1.906	21.8	0.057
Class 4 referred to class 1					
IES	0.163	0.031	5.324	17.7	0.000
ISI	0.187	0.047	3.951	20.6	0.000
PHQ	0.348	0.08	4.373	41.6	0.000
GAD	0.184	0.11	1.671	20.2	0.095
Class 3 referred to class 2					
IES	0.064	0.012	5.548	6.6	0.000
ISI	0.101	0.029	3.505	10.6	0.000
PHQ	0.072	0.043	1.681	7.5	0.093
GAD	−0.05	0.05	−1.007	−4.9	0.314
Class 4 referred to class 2					
IES	0.047	0.014	3.379	4.8	0.001
ISI	0.149	0.033	4.454	16.1	0.000
PHQ	0.225	0.056	4.048	25.2	0.000
GAD	−0.063	0.06	−1.054	−6.1	0.292
Class 4 referred to class 3					
IES	−0.017	0.012	−1.497	−1.7	0.134
ISI	0.048	0.025	1.909	4.9	0.056
PHQ	0.153	0.049	3.127	16.5	0.002
GAD	−0.013	0.052	−0.248	−1.3	0.804

IES, The impact of Event Scale-revised; ISI, Insomnia Severity Index; PHQ, Patient Health Questionnaire items; GAD, The Generalized Anxiety Disorder Questionnaire; Est, estimation; s.e., standard error.

## Discussion

This study examined the extent to which psychological outcomes of a pandemic were associated with risk perceptions in a sample of nurses working against COVID-19 using an LPA approach. We identified four subgroups of nurses: a low-level class, a mild-level class, a moderate-level class, and a high-level class for all risk perceptions. Anxiety elevated nurses' risk perception to a mild level, while the PTSD after COVID-19, insomnia, and depression would accelerate the process, reaching a moderate and high level of risk perceptions. Overall, a poor psychological status confirmed by the clinic questionnaires could predict a high level of risk perceptions to nursing works.

These findings are consistent with previous studies that found a link between negative emotions, sleep problems, PTSD, and risk perceptions (Frings, 2012; Spada and Reisse, 2012; Palgi *et al*., 2018; Chew *et al*., 2020). As a consensus, a high level of perceived risk could have a side-effect on the mental health of people during public health crises (Ding *et al*., 2020). However, these studies did not report recurrences of mental problems caused by the crisis without a follow-up study in the back-to-normal process. In particular, our study was the first to explore the post-acute sequelae of COVID-19 in the mental health of nurses and the risk perception for their work. Although without a clinical diagnosis and based on the participants' self-reported possibilities and severities of mental problems, our results still proved that psychological outcomes of COVID-19 elevated the level of nurses' risk perceptions, supported by the fact that the members in class 4 (showing a high level of risk perceptions) have a high prevalence of PTSD, sleep, and emotional disorders.

Additionally, in our cohort, PTSD, insomnia, depression, and anxiety were not as severe as previous studies reported immediately after the outbreak of COVID-19 given their total scores on the subscales (Magnavita *et al*., 2020; Tan *et al*., 2020; Yin *et al*., 2020), while a series of studies conducted at different stages of COVID-19 in Central Italy showed that the psychological problems of health care workers were not significantly improved and that various problems and worries were perceived in their work and life (Magnavita *et al*., 2021*a*, 2021*b*). Moreover, a recent review also reported that mental health issues, such as anxiety, depression, PTSD, and sleep disorders, had great impacts on health care workers, especially on their contacts with the public (Giorgi *et al*., 2020). Therefore, we cautiously interpreted our findings with studies after COVID-19. Most nurses stimulated their worries about their physical health risk and time pressures from their works signaled by anxiety, which could be an adaptive self-protective reaction to the crisis. However, depression, insomnia, and PTSD, as worse by-products of COVID-19, could continue to deteriorate and elevate nurses' risk perceptions of fears, avoidance, and discouragement. The persistent debate over COVID-19 would discombobulate nurses when they encounter risk. Worse, these nurses would not seek the help of a mental health professional as they lack the energy and inclination to do so. Therefore, there could be no positive changes in the prevalence of depression, insomnia, and PTSD. Miserably, suicide and substance use disorders could be further problems (Valente, 2011).

To prevent tragedies, we must acknowledge that this pandemic has created enormous stress and heartache in our health care professionals, especially nurse clinicians, despite their attempts to mask it. Without the lessons taught by the COVID-19 pandemic, few could have imagined or anticipated a situation such as this. Hence, special attention should be given to nurses, especially those who avoid clinical practices. Psychological programs to relieve anxiety and fear such as mental exercises and gradual desensitization should be designed to intervene in psychological reactions and risk perceptions. Furthermore, although nurses are praised for their heroic actions during the COVID-19 pandemic to shoulder their responsibilities and spare no efforts to guard public health, which are undoubtedly appreciated, we must be cautious that heroic actions – voluntary prosocial actions – associated with an acknowledged degree of personal risk, transcend the duty of nurses, and aggregate their psychosocial risks (MacDonald *et al*., 2018). After the outbreak of COVID-19, more discussions centered around the risk and obligation of ‘duty of care’, or ‘duty to treat’, weighing the risks against their duties (WHO, 2020*b*). Consequently, the prevalence of psychosis has been emphasized by many studies. However, fragments of psychological help are not timely and lack follow-up; therefore, at the latter stage of the pandemic, the psychological outcomes were predominantly reflected in risk perceptions for work and life (Kontoangelos *et al*., 2020; Saladino *et al*., 2020). Nurses’ feelings of devoting less time to physical activity or meditation and intellectual activities reduce resilience and hinder the application of individual psychological support treatments. Perceived heavy workload could be alleviated by increasing staff, but adequately trained clinic personnel are not available given that fewer nurses are willing to practice in the clinics. Therefore, interventions on nurses' risk perceptions through ameliorating the psychological impact of the COVID-19 pandemic are imperative. It should be a network to ease all factors for overly elevated risk perceptions to motivate nurses. Clinicians who provide care for moral injury and associated mental illness should also be aware of opportunities to speak about the worries of their jobs and focus on their psychological status during therapy. Supervisors should ensure that time is allotted to reflect on and learn from extraordinarily difficult experiences and encourage therapeutic intervention. Additionally, nurses themselves should increase their knowledge about their work and not be shameful about their worries and fears; instead, nurses should discuss their concerns with peers who are experiencing similar concerns.

### Limitations

There are limitations to the conclusions that can be drawn from this study. As mentioned, LPA is a data-determined statistical approach. The analysis described in this paper was undertaken using a sample of five hospital nurses, in Jiangsu, China. A strength of our sample is that it was collected from a place where there were no other emergencies except COVID-19. However, there was no significant disparity of exposures in our cohort; therefore, findings generalized to other samples await further investigation. Additionally, the analysis was cross-sectional, and it was not possible to determine a cause-effect relationship. Finally, a limitation inherent in LPA is that it classifies individuals into distinct subclasses based on the dichotomization of constructs. However, an advantage of the approach is that it enables researchers to sort individuals into relatively homogeneous subgroups that are more similar to each other than other subgroups within a sample (Borsboom *et al*., 2016). A similar application was also seen in the study of public people with various levels of risk perceptions (Wang *et al*., 2021). In our study, the technique helps evaluate the characteristics of nurses' risk perceptions through the application of varied statistical approaches.

Certainly, further insights can be gained from the ongoing analysis of the factors that promote nurses' willingness to work. For example, risk communication, referring to exchanging information, advice, and opinions, enables at-risk people to make judgements of uncertainty and to protect themselves. The frequency of risk communications between nurses and patients, doctors, and supervisors creates the foundation for social support systems, which directly affects the individual psychological state and perceptions (Ell, 1984). Another study of our cohort also found a buffer effect of perceived social support on anxiety 1 year after the COVID-19 (Hou *et al*., 2021). However, it is unknown about how nurses' risk perceptions coevolve with social connections. The role of risk communication in various classes of risk perceptions and psychological outcomes after the COVID-19 pandemic warrants further study. Hence, it is necessary to examine the roles of the individual, psychosocial, and other structural variables in various classes of risk perceptions and psychological outcomes. Notably, longitudinal studies with proper intervention may contribute to confirming previous findings and inspiring future research.

## Conclusion

There are extraordinary times during the COVID-19 pandemic. Now, there is an urgent need to ensure that the tasks ahead do not cause long-lasting damage to health care staff. The results of this study, consistent with theoretical conceptions, highlight the important impacts of psychological outcomes after the outbreak of COVID-19 on risk perceptions among nurses. As an appropriate job-related risk perception is closely related to nurses working motivation, it is imperative to intervene in nurses' psychological outcomes, such as PTSD, insomnia, depression, and other server outcomes, while simultaneously focusing on work-related worries.

## Data

The data that support the findings of this study are available upon reasonable request.

## References

[ref1] Aakko E (2004) Risk communication, risk perception, and public health. WMJ Official Publication of the State Medical Society of Wisconsin 103, 25–27. 15101463

[ref2] Asparouhov T and Muthén B (2014) Auxiliary variables in mixture modeling: three-step approaches using M plus. Structural Equation Modeling: a Multidisciplinary Journal 21, 329–341.

[ref3] Bastien CH, Vallières A and Morin CM (2001) Validation of the Insomnia Severity Index as an outcome measure for insomnia research. Sleep Medicine 2, 297–307. 11438246 10.1016/s1389-9457(00)00065-4

[ref4] Bauer DJ and Curran PJ (2003) Distributional assumptions of growth mixture models: implications for overextraction of latent trajectory classes. Psychological Methods 8, 338–363. 14596495 10.1037/1082-989X.8.3.338

[ref5] Borsboom D, Rhemtulla M, Cramer AOJ, Van der Maas HLJ, Scheffer M and Dolan CV (2016) Kinds versus continua: a review of psychometric approaches to uncover the structure of psychiatric constructs. Psychological Medicine 46, 1567–1579. 26997244 10.1017/S0033291715001944

[ref6] Chaffee M (2009) Willingness of health care personnel to work in a disaster: an integrative review of the literature. Disaster Medicine Public Health Preparedness 3, 42–56. 19293743 10.1097/DMP.0b013e31818e8934

[ref7] Chaiklin H (1987) Unraveling the mystery of health: how people manage stress and stay well. Journal of Nervous Mental Disease 177, 439–440.

[ref8] Champion VL and Skinner CS (2008) The Health Belief Model. San Francisco, CA: John Wiley & Sons.

[ref9] Chew NW, Lee GK, Tan BY, Jing M, Goh Y, Ngiam NJ, Yeo LL, Ahmad A, Khan FA and Shanmugam GN (2020) A multinational, multicentre study on the psychological outcomes and associated physical symptoms amongst healthcare workers during COVID-19 outbreak. Brain, Behavior, Immunity 88, 559–565. 32330593 10.1016/j.bbi.2020.04.049 PMC7172854

[ref10] Chiang HH, Chen MB and Sue IL (2007) Self-state of nurses in caring for SARS survivors. Nursing Ethics 14, 18. 17334167 10.1177/0969733007071353

[ref11] Christianson S and Marren J (2012) The impact of event scale-revised (IES-R). Medsurg Nursing 21, 321–322. 23243796

[ref12] Cornelia B, Wieler LH and Katrine H (2020) Monitoring behavioural insights related to COVID-19. Lancet 395, 1255–1256. 32247323 10.1016/S0140-6736(20)30729-7 PMC7163179

[ref13] Ding Y, Xu J, Huang S, Li P, Lu C and Xie S (2020) Risk perception and depression in public health crises: evidence from the COVID-19 crisis in China. International Journal of Environmental Research and Public Health 17, 5728. 32784792 10.3390/ijerph17165728 PMC7460398

[ref14] Ell K (1984) Social networks, social support, and health status: a review. Social Service Review 58, 133–149.

[ref15] Frings D (2012) The effects of sleep debt on risk perception, risk attraction and betting behavior during a blackjack style gambling task. Journal of Gambling Studies 28, 393–403. 21915653 10.1007/s10899-011-9266-9

[ref16] Giorgi G, Lecca LI, Alessio F, Finstad GL, Bondanini G, Lulli LG, Arcangeli G and Mucci N (2020) COVID-19-related mental health effects in the workplace: a narrative review. International Journal of Environmental Research and Public Health 17, 21. 10.3390/ijerph17217857 PMC7663773 33120930

[ref17] Grimm K (2017) Multilevel modeling using Mplus. A Multidisciplinary Journal 24, 960–962.

[ref18] Hagenaars JA and Mccutcheon AL (2002) Applied Latent Class Analysis. New York, NY: Cambridge University Press.

[ref19] He XY, Li CB, Qian J, Cui HS and Wu WY (2010) Reliability and validity of a generalized anxiety disorder scale in general hospital outpatient. Shanghai Archives of Psychiatry 22, 200–203.

[ref20] Homko CJ, Santamore WP, Zamora L, Shirk G, Gaughan J, Cross R, Kashem A, Petersen S and Bove AA (2008) Cardiovascular disease knowledge and risk perception among underserved individuals at increased risk of cardiovascular disease. Journal of Cardiovascular Nursing 23, 332–337. 18596496 10.1097/01.JCN.0000317432.44586.aa

[ref21] Hope K, Massey PD, Osbourn M, Durrheim DN and Turner C (2011) Senior clinical nurses effectively contribute to the pandemic public health response. Australian Journal of Advanced Nursing A Quarterly Publication of the Royal Australian Nursing Federation 28, 47–53.

[ref22] Hou T, Yin Q, Xu Y, Gao J, Bin L, Li H, Cai W, Liu Y, Dong W, Deng G and Ni C (2021) The mediating role of perceived social support between resilience and anxiety 1 year after the COVID-19 pandemic: disparity between high-risk and low-risk nurses in China. Frontiers in Psychiatry 12, 666789. 34108897 10.3389/fpsyt.2021.666789 PMC8180566

[ref23] Janz NK and Becker MH (1984) The health belief model: a decade later. Health Education Quarterly 11, 1–47. 6392204 10.1177/109019818401100101

[ref24] Kendra MA and George VD (2010) Defining risk in home visiting. Public Health Nursing 18, 128–137. 10.1046/j.1525-1446.2001.00128.x 11285107

[ref25] Khalid M, Khalid H, Bhimani S, Bhimani S, Khan S, Choudry E and Mahmood SU (2021) Risk perception and willingness to work among doctors and medical students of Karachi, Pakistan during the COVID-19 pandemic: a web-based cross-sectional survey. Risk Management Healthcare Policy 14, 3265. 34408512 10.2147/RMHP.S310453 PMC8364387

[ref26] Koh Y, Hegney DG and Drury V (2011) Comprehensive systematic review of healthcare workers’ perceptions of risk and use of coping strategies towards emerging respiratory infectious diseases. International Journal of Evidence-Based Healthcare 9, 403–419. 22093389 10.1111/j.1744-1609.2011.00242.x

[ref27] Koh Y, Hegney D and Drury V (2012) Nurses’ perceptions of risk from emerging respiratory infectious diseases: a Singapore study. International Journal of Nursing Practice 18, 195–204. 22435984 10.1111/j.1440-172X.2012.02018.x PMC7165875

[ref28] Kontoangelos K, Economou M and Papageorgiou C (2020) Mental health effects of COVID-19 pandemic: a review of clinical and psychological traits. Psychiatry Investigation 17, 491. 32570296 10.30773/pi.2020.0161 PMC7324731

[ref29] Kroenke K, Spitzer RL and Williams JBW (2001) The PHQ-9: validity of a brief depression severity measure. Journal of General Internal Medicine 16, 606–613. 11556941 10.1046/j.1525-1497.2001.016009606.x PMC1495268

[ref30] Linden SVD (2014) On the relationship between personal experience, affect and risk perception: the case of climate change. European Journal of Social Psychology 44, 430–440. 25678723 10.1002/ejsp.2008 PMC4312984

[ref31] Linden SVD (2015) The social-psychological determinants of climate change risk perceptions: towards a comprehensive model. Journal of Environmental Psychology 41, 112–124.

[ref32] MacDonald K, De Zylva J, McAllister M and Brien DL (2018) Heroism and nursing: a thematic review of the literature. Nurse Education Today 68, 134–140. 29908409 10.1016/j.nedt.2018.06.004

[ref33] Magnavita N, Tripepi G and Prinzio RRD (2020) Symptoms in health care workers during the COVID-19 epidemic. A cross-sectional survey. International Journal of Environmental Research and Public Health 17, 5218. 32698320 10.3390/ijerph17145218 PMC7400440

[ref34] Magnavita N, Soave PM and Antonelli M (2021 *a*) Prolonged stress causes depression in frontline workers facing the COVID-19 pandemic – a repeated cross-sectional study in a COVID-19 hub-hospital in central Italy. International Journal of Environmental Research and Public Health 18, 7316. 34299767 10.3390/ijerph18147316 PMC8304927

[ref35] Magnavita N, Soave PM and Antonelli M (2021 *b*) One-year prospective study of occupational health in the intensivists of a COVID-19 hub hospital. International Journal of Environmental Research and Public Health 18, 9888. 34574811 10.3390/ijerph18189888 PMC8466101

[ref36] Nylund KL, Asparouhov T and Muthén BO (2007) Deciding on the number of classes in latent class analysis and growth mixture modeling: a monte Carlo simulation study. Structural Equation Modeling A Multidisciplinary Journal 14, 535–569.

[ref37] Osofsky JD, Osofsky HJ and Mamon LY (2020) Psychological and social impact of COVID-19. Psychological Trauma: Theory, Research, Practice, Policy 12, 468. 32538653 10.1037/tra0000656

[ref38] Palgi Y, Avidor S, Shrira A, Bodner E, Ben-Ezra M, Zaslavsky O and Hoffman Y (2018) Perception counts: the relationships of inner perceptions of trauma and PTSD symptoms across time. Psychiatry Clinical Neurosciences 81, 361–375. 10.1080/00332747.2018.1485370 30216131

[ref39] Que J, Shi L, Deng J, Liu J, Zhang L, Wu S, Gong Y, Huang W, Yuan K and Yan W (2020) Psychological impact of the COVID-19 pandemic on healthcare workers: a cross-sectional study in China. General Psychiatry 33, e100259. 32596640 10.1136/gpsych-2020-100259 PMC7299004

[ref40] Radtke JV, Tate JA and Happ MB (2012) Nurses’ perceptions of communication training in the ICU. Intensive Critical Care Nursing 28, 16–25. 22172745 10.1016/j.iccn.2011.11.005 PMC3264744

[ref41] Risti DI, Hini D, Bankovi D, Koovi A and Gavrilovi J (2020) Levels of stress and resilience related to the COVID pandemic among academic medical staff in Serbia. Psychiatry Clinical Neurosciences 74, 604–605. 32738004 10.1111/pcn.13124 PMC7436760

[ref42] Rosenstock IM (1974) Historical origins of the health belief model. Health Education Monographs 2, 328–335. 10.1177/109019817800600406 299611

[ref43] Saladino V, Algeri D and Auriemma V (2020) The psychological and social impact of Covid-19: new perspectives of well-being. Frontiers in Psychology 11, 2550. 10.3389/fpsyg.2020.577684 PMC7561673 33132986

[ref44] Samadipour E, Ghardashi F and Aghaei N (2020) Evaluation of risk perception of Covid-19 disease: a community-based participatory study. Disaster Medicine Public Health Preparedness 3, 1–8. 10.1017/dmp.2020.311 PMC7642912 32873355

[ref45] Santamaria MD, Jauregizar J, Redondo I, Etxebarria NO and Picaza M (2020) Psychological symptoms in health professionals in Spain after the first wave of the COVID-19 pandemic. Frontiers in Physiology 11, 3400. 10.3389/fpsyg.2020.606121 PMC7775406 33391125

[ref46] Seale H, Leask J, PoC K and MacIntyre R (2009) Will they just pack up and leave? Attitudes and intended behaviour of hospital health care workers during an influenza pandemic. Bmc Health Services Research 9, 1–8. 19216792 10.1186/1472-6963-9-30 PMC2661074

[ref47] Shirey MR (2010) Nurse manager cognitive decision-making. Nursing Annual Communicating Nursing Research Conference, Western.

[ref48] Skarl S (2015) Anxiety and depression association of America. Journal of Consumer Health on the Internet 19, 100–106.

[ref49] Spada H and Reisse K (2012) Cognition and emotion in risk perception and behavior – security awareness/need for security. Resilienz in der offenen Gesellschaft,.

[ref50] Standing M (2010) Clinical judgement and decision-making in nursing – nine modes of practice in a revised cognitive continuum. Journal of Advanced Nursing 62, 124–134. 10.1111/j.1365-2648.2007.04583.x 18352971

[ref51] Tan BY, Chew NWS, Lee GKH, Jing M and Sharma VK (2020) Psychological impact of the COVID-19 pandemic on health care workers in Singapore. Annals of Internal Medicine 173, 317–320. 32251513 10.7326/M20-1083 PMC7143149

[ref52] Valente S (2011) Nurses’ psychosocial barriers to suicide risk management. Nursing Research 2011, 4. 10.1155/2011/650765 PMC3169808 21994837

[ref53] Vermunt JK (2010) Latent class modeling with covariates: two improved three-step approaches. Political Analysis 18, 450–469.

[ref54] Wang P-W, Chen Y-L, Chang Y-P, Wu C-F, Lu W-H and Yen C-F (2021) Sources of COVID-19-related information in people with various levels of risk perception and preventive behaviors in Taiwan: a latent profile analysis. International Journal of Environmental Research and Public Health 18, 2091. 33669977 10.3390/ijerph18042091 PMC7924873

[ref55] World Health Organization (2020 *a*) *How contributions support WHO's work in ongoing fight of COVID-*19 *pandemic around the world*. World Health Organization. Retrieved 22 January from.

[ref56] World Health Organization (2020 *b*) Pandemic fatigue: reinvigorating the public to prevent COVID-19: policy considerations for member states in the WHO European region. R. O. f. Europe. Available at https://apps.who.int/iris/handle/10665/335820.

[ref57] Xie J, Tong Z, Guan X, Du B, Qiu H and Slutsky AS (2020) Critical care crisis and some recommendations during the COVID-19 epidemic in China. Intensive Care Medicine 46, 837–840. 32123994 10.1007/s00134-020-05979-7 PMC7080165

[ref58] Yancy CW (2020) COVID-19 and African Americans. JAMA 323, 1891–1892. 32293639 10.1001/jama.2020.6548

[ref59] Yin Q, Sun Z, Liu T, Ni X, Deng X, Jia Y, Shang Z, Zhou Y and Liu W (2020) Posttraumatic stress symptoms of health care workers during the corona virus disease 2019. Clinical Psychology & Psychotherapy 27, 384–395. 32415733 10.1002/cpp.2477 PMC7276761

[ref60] Zhang X, Cao G, Xu Z, Chen Z, Zhang Y and Cao B (2016) Compliation of the questionnaire for nurses' risk perceptioin. Nursing research 30, 2353–2355.

